# Small Bowel Perforation Secondary to Esophageal Stent Migration: A Comparative Review of Six Cases

**DOI:** 10.7759/cureus.3455

**Published:** 2018-10-16

**Authors:** Syed H Tasleem, Faisal Inayat, Nouman Safdar Ali, Saud Bin Abdul Sattar, Ahmed Munir, Fahad Zafar

**Affiliations:** 1 Gastroenterology and Hepatology, Baylor College of Medicine, Houston, USA; 2 Internal Medicine, Allama Iqbal Medical College, Lahore, PAK; 3 Internal Medicine, Services Institute of Medical Sciences, Lahore, PAK; 4 Internal Medicine, King Edward Medical University, Lahore, PAK

**Keywords:** small bowel perforation, esophageal stent migration, stent complications, management, endoscopic intervention

## Abstract

Esophageal stent placement is used to treat benign strictures, esophageal perforations, fistulas and for palliative therapy of esophageal cancer. Although it is a safe and effective method, complications are increasing the morbidity and mortality rate. Small bowel perforation as a result of esophageal stent migration is a remarkably rare occurrence. We report one case from our clinical experience and undertake a review of the previously reported cases retrieved from the PubMed. A total of six cases were found accessible. Abdominal pain was the common clinical presentation. The mean time from stent placement to perforation was 3.4 months (range, two weeks to 12 months). The jejunum was the frequently perforated portion of the small bowel. Surgical intervention was the mainstay of treatment. This comparative review illustrates that clinicians should remain vigilant for small bowel perforation in patients with esophageal stent placement. Further studies are required to delineate the magnitude and scope of this association.

## Introduction

Endoscopic stent placement has frequently been used to maintain the esophageal luminal patency in patients with strictures, esophageal perforations, fistulas, and for palliative treatment of esophageal cancer [1, 2]. However, various immediate and delayed complications associated with this procedure are relatively increasing the morbidity and mortality rate. Stent migration is among the most commonly encountered problems in these patients [3, 4]. In one survey, it occurred in 6.8% of 434 patients who underwent esophageal stent placement [5]. It is notable that most migrations are often asymptomatic. However, serious complications such as tracheoesophageal fistula, hemorrhage, obstruction and rarely, gastrointestinal perforation may also occur. Small bowel perforation as a result of esophageal stent migration is a rare but high-risk clinicopathologic entity with only a handful of cases reported thus far [5]. Herein, we chronicle the case of a patient where esophageal self-expanding metallic stent migration culminated in a jejunal perforation. Furthermore, this review outlines our current understanding of the epidemiology of and risk factors for esophageal stent migration-related small bowel perforation, the pathophysiology of this condition and currently available approaches to diagnosis and treatment.

## Case presentation

A 64-year-old female presented to our medical center with abdominal pain, nausea, and vomiting for one day. The pain was diffuse, sharp, and it was concentrated in the lower abdomen. Her past medical history was significant for breast carcinoma status post bilateral mastectomy, hysterectomy, *Helicobacter pylori* infection successfully treated with triple therapy and metastatic squamous cell carcinoma of the esophagus. Four years ago, she underwent an uneventful placement of a fully covered 19 x 100-mm esophageal Wallflex® stent (Boston Scientific, Natick, MA, USA). Her family history was unremarkable and her home medications included iron and vitamin C supplementation. The patient was non-alcoholic, non-smoker and drug-free. On physical examination, she appeared comfortable; well-oriented in time, space and person; well nourished; and there was no acute distress. Cardiac examination was notable for tachycardia. The chest was clear to auscultation with good air entry bilaterally. The abdomen was tender in the hypogastrium and left-lower quadrant, but it was soft and non-distended. Vital sign examination revealed blood pressure 112/68 mm Hg, heart rate 111 beats per minute, temperature 98.5°F and respiratory rate of 18 breaths per minute.

The patient underwent an extensive diagnostic evaluation. The details of her laboratory workup are provided in Table 1.

**Table 1:** Initial laboratory investigations of the patient with respective reference ranges. AST: Aspartate aminotransferase; ALT: Alanine aminotransferase; ALP: Alkaline phosphatase.

Laboratory parameter	Specimen	Patient result	Reference range
Hemoglobin	Serum	10	13-18 g/dL
Hematocrit	Serum	30.3	40%-52%
White blood cells	Serum	28.7	4.5-11.0/uL
Platelets	Serum	250 x 10^3^	150-450 x 10^3^/uL
Blood urea nitrogen	Serum	26	6-22 mg/dL
Creatinine	Serum	1.79	0.4-1.2 mg/dL
Sodium	Serum	136	135-145 mmol/L
Potassium	Serum	5.1	3.5-5.0 mmol/L
AST	Serum	35	5-40 U/L
ALT	Serum	23	7-56 U/L
ALP	Serum	143	44-147 U/L
Total bilirubin	Serum	0.90	0.1-1.2 mg/dL

**Figure 1:**
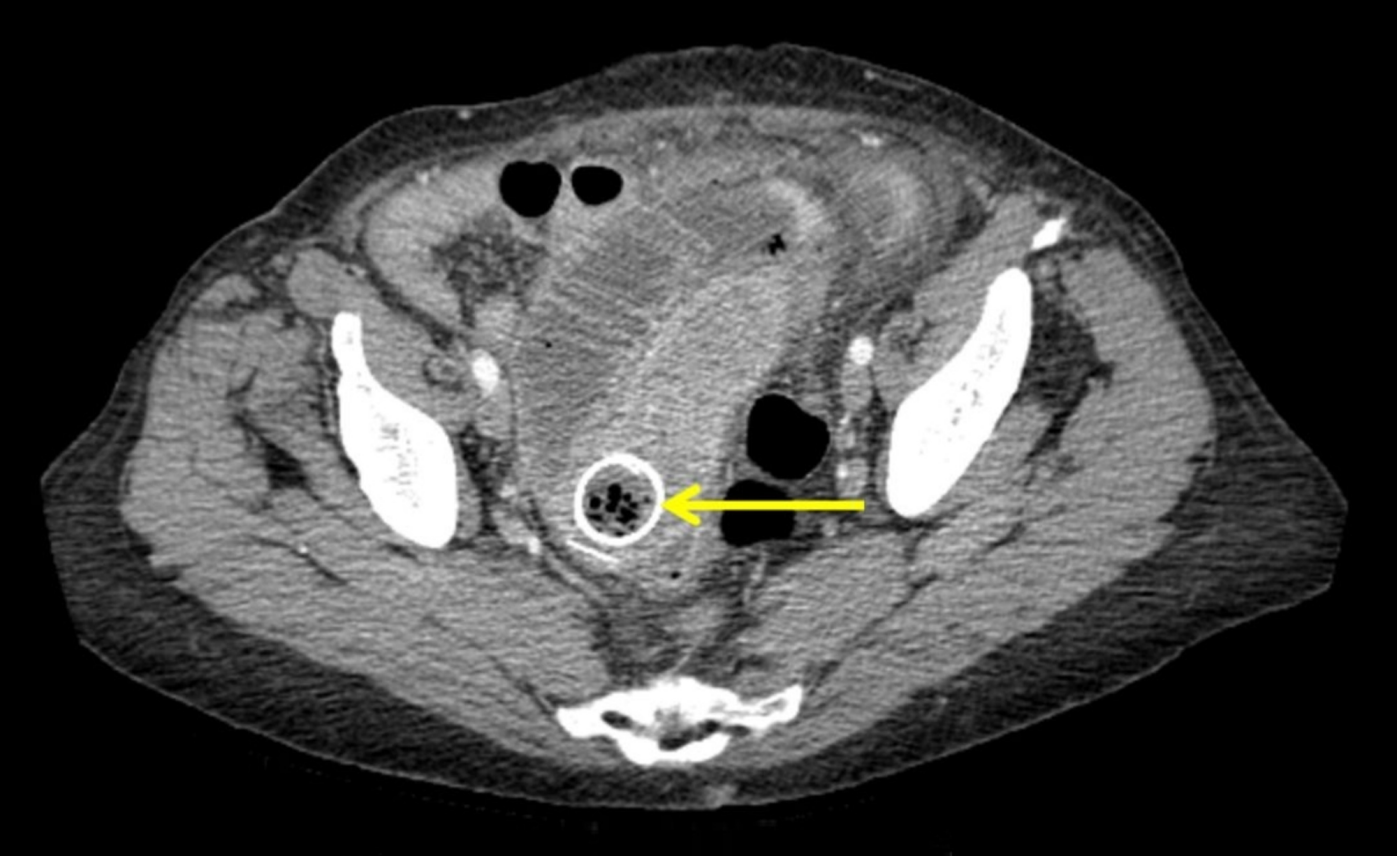
Computed tomography abdomen showing migrated stent located in the left-lower quadrant of the abdomen causing obstruction with the presence of free air in the abdominal cavity (Axial view). Arrow indicates the precise location of the stent.

Computed tomography abdomen identified the esophageal stent in the left-lower quadrant of the abdomen with the presence of free air in the abdominal cavity, consistent with the gastrointestinal tract perforation (Figure 1).

**Figure 2:**
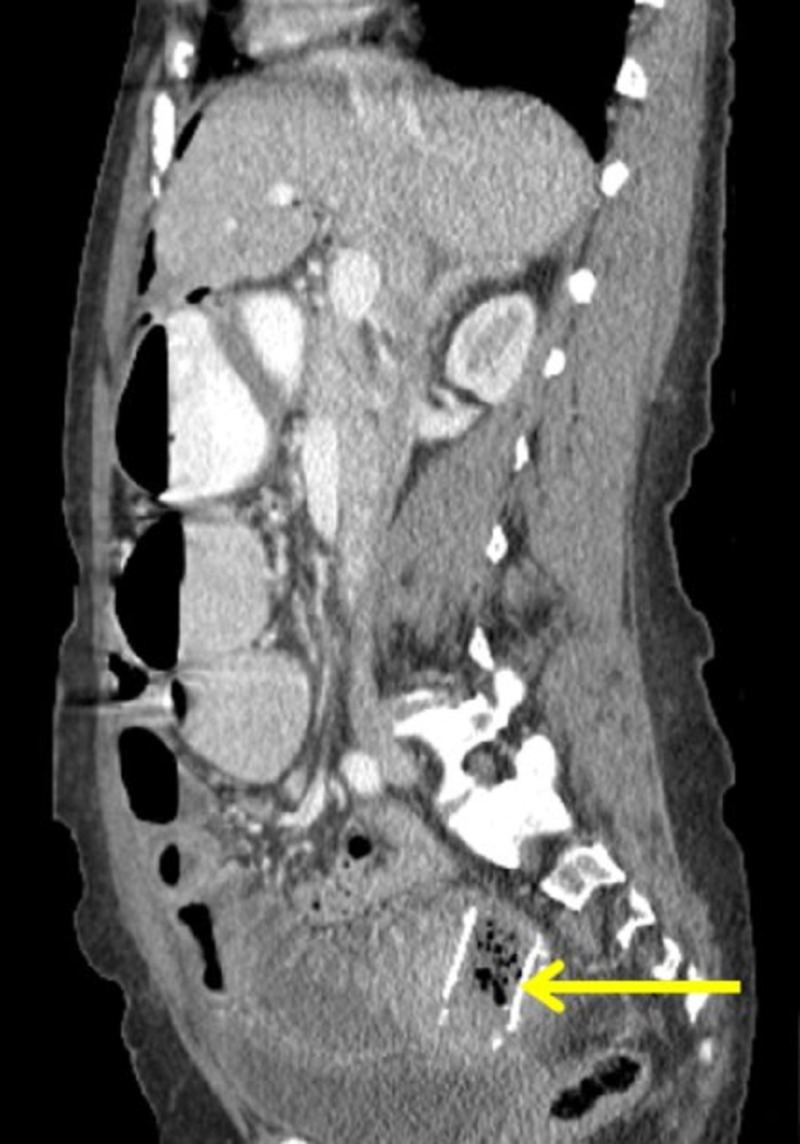
Computed tomography abdomen showing esophageal migrated stent-related perforation of the jejunal part of the small bowel; multiple loops of small bowel measuring up to 4.5 cm were noted (Coronal view). Arrow demarcates the location of the migrated stent.

The migrated stent-related perforation of the jejunum with multiple loops of small bowel measuring up to 4.5 cm, bowel-wall edema, and thickening of the loops proximal to the stent were evident (Figure 2).

The patient was emergently shifted to an operating room. An uneventful exploratory laparotomy was performed. It showed a moderate collection of a purulent fluid within the abdomen. The esophageal stent was palpated 16 cm into the jejunum in the pelvis, with markedly dilated proximal small bowel. The stent along with the perforated distal jejunum was resected and an end-to-end anastomosis was performed. The post-procedure clinical course of the patient was unremarkable. After she resumed enteral feeds, she was discharged from the hospital in stable condition. On the one-month follow-up, she reported a good recovery without any inadvertent events or recurrence of the gastrointestinal complaints.

## Discussion

Esophageal stent placement is relatively a safe treatment for esophageal luminal compromise, regardless of the underlying disease [6]. The common indications of this procedure include benign or malignant stenosis, perforation, fistula, leak, stricture, or a palliative procedure in patients with esophageal cancer. Similarly, several surgical complications of procedures like esophagectomy, gastric sleeve, gastrectomy, and gastric bypass may also warrant stenting [6]. The stent anchors to the esophageal walls by self-expansion. It provides adequate functionality and enhances the quality of life, especially in patients with incurable esophageal cancer. In these patients, the inevitable consequences of progressive esophageal tumors can be delayed and/or averted with a timely stenting [7]. It has been observed that up to 95% of the patients with esophageal stent placement were at least able to tolerate liquid intake after the procedure [8]. However, esophageal stenting has also been associated with a constellation of complications.

We performed an extensive literature search of the medical database, PubMed (National Library of Medicine, Bethesda, MD). Search criteria comprised of a combination of terms, including “esophagus”, “stent”, “esophageal stent placement” and “small bowel perforation” that helped to retrieve several accessible publications. After a careful review of all the search results, we identified a total of six cases with small bowel perforation secondary to esophageal stent migration reported as of September 2018 [9-14]. The data on patients’ characteristics, epidemiology, clinical features, medical diagnosis, the time interval for the event, the portion of small bowel involved, and management are summarized in Table 2.

**Table 2:** Literature review of small bowel perforation due to esophageal stent migration. SCC-E: Squamous cell carcinoma of esophagus; EAD: Esophageal adenocarcinoma; TEF: Tracheoesophageal fistula.

Authors	Age/ gender/ country	Clinical presentation	Diagnosis	Perforation time after stenting	Perforation site	Probable risk factors	Treatment	Outcome
Henne et al. 1997 [9]	52/F/ Germany	Abdominal pain	SCC-E	Two weeks	Jejunum	Prior esophagogastrectomy, multiple stents	Segmental bowel resection	Recovered
Kim et al. 2000 [10]	86/M/ Korea	Abdominal pain, nausea, vomiting	SCC-E	Two months	Duodenum	Obligatory stent position and extrinsic bowel fixation	Percutaneous drainage	Recovered
Reddy et al. 2009 [11]	79/F/UK	Abdominal pain, nausea	SCC-E	12 months	Ileum	Stent fracture, decrease in tumor size due to chemo/radiotherapy	Right hemicolectomy	Recovered
Bay and Penninga, 2010 [12]	80/M/ Denmark	Abdominal pain, nausea	EAD	Three months	Jejunum	Decrease in tumor size due to chemo/radiotherapy	Segmental bowel resection	Recovered
Zhang et al. 2011 [13]	17/M/ China	Abdominal pain, nausea	TEF	Three weeks	Jejunum	Proximal jejunal fixation to the ligament of Treitz	Perforation closure, anal expulsion of stent	Recovered
Karagul et al. 2015 [14]	77/M/ Turkey	Abdominal pain, vomiting, distension	EAD	Two months	Ileum	Esophagectomy	Segmental bowel resection	Recovered
The present report	64/F/ USA	Abdominal pain, nausea, vomiting	SCC-E	48 months	Jejunum	Decrease in tumor size due to chemo/radiotherapy	Segmental bowel resection	Recovered

In this review, the average age of the patients was 65 years (range: 17-86 years). Abdominal pain was the typical clinical presentation that was mostly associated with nausea and vomiting. Occasionally, abdominal distension with generalized tenderness and rebound were also noted. In the current review, esophageal squamous cell carcinoma (n = 3), esophageal adenocarcinoma (n = 2), and tracheoesophageal fistula (n = 1) were among the indications of stenting. The time period between initial stent placement and small bowel perforation was highly variable, ranging from two weeks to 12 months. In our patient, the symptoms of migrated stent-related perforation presented after 48 months. The commonest site of perforation was jejunum (n = 3) followed by ileum (n = 2) and duodenum (n = 1).

The exact pathogenesis of esophageal stent migration is unknown. The stent usually relies on the adhesive force and frictional resistance between its body and the walls of the esophagus [15]. Tumor debulking by chemotherapy and radiotherapy as well as the use of fully-covered and plastic stents can reduce friction between stent and esophageal wall that may lead to migration. The diameter of stents is another important factor in this regard. The metallic stents with a high ratio of expanded diameter to introduction diameter are less prone to migration as they adhere strongly to the gastrointestinal walls. Other probable risk factors for migration include stents with smaller diameters, stent fracture, esophageal peristalsis and anatomical changes in the gastrointestinal tract such as resection of gastroesophageal junction [16]. In a vast majority of the cases, esophageal stents migrate no farther than the stomach that potentially incurs a low probability of complications. Although this phenomenon is extremely rare, the migrated stents may lead to small bowel perforation by mucosal impaction, erosion and/or ulceration [17]. In this review, prior esophagogastrectomy, concomitant use of multiple stents, obligatory stent position, extrinsic bowel fixation, debulking of the tumor with chemotherapy and radiations, stent fractures, and proximal jejunal fixation were among the plausible risk factors that contributed to the stent migration. Recently, a number of new stent-fixation procedures are investigated to prevent the migration [17]. However, further research is warranted in this regard, especially to avoid devastating complications like intestinal perforation.

In regard to diagnosis, acquiring a detailed clinical history and focused physical examination are pertinent. A timely exclusion of bowel obstruction and perforation, especially in patients presenting to the emergency department with severe, sudden-onset gastrointestinal symptoms is of paramount importance. In most cases, the dislodged stent cannot be detected early in the course of the disease. The initial blood tests should include a complete biochemical profile, liver function testing, pancreatic enzymes, and serum electrolytes in order to assess the clinical status of the patient and to rule out other possible etiologies [17]. In cases with suspected perforation, a prompt abdominal radiograph is imperative to exclude pneumoperitoneum and to assess location of the dislodged stent. However, a contrast-enhanced computed tomography scan of the abdomen and pelvis is the most important investigation in these patients that demonstrates a high diagnostic yield [17]. Furthermore, endoscopy can also be performed to identify and retrieve the dislodged stents in difficult-to-diagnose cases.

Although a standard treatment approach is not available, initial clinical presentation is critical for therapeutic decision making. After conservative management in hemodynamically stable patients, the determination of the precise location of the migrated stent is essentially important in order to decide about the surgical or non-surgical approach. Most esophageal stents do not migrate farther than the stomach. Therefore, a majority of patients are asymptomatic and migrated stent may remain in the stomach without complications. Due to the flexible nature, most of the migrated esophageal stents can be impacted in the stomach for a long time and subsequently, they can evacuate through the anal route without any inadvertent events [18]. Therefore, intervention can be delayed in asymptomatic patients with serial radiologic assessment.

Endoscopic or surgical removal of the migrated stent is warranted in symptomatic patients. Therapeutic endoscopy is preferable in cases with no signs of intestinal obstruction or perforation [18]. It is performed by using grasping forceps, polypectomy snare, and balloon dilatation catheters utilizing the expertise of an experienced endoscopist. The incompletely migrated stent can be picked up endoscopically by using forceps and it is pulled up to its normal position where it is fixed with suturing to reduce the risk of migration in future. In cases where endoscopic retrieval fails, the symptoms of total gastric stent migration can be alleviated by gastrotomy [19]. However, surgical removal of the stent along with the affected portion of the bowel followed by end-to-end anastomosis is the mainstay of management if endoscopic retrieval is unsuccessful or contraindicated, especially in patients with bowel perforation [20]. In our review, most patients underwent segmental bowel resection. Percutaneous drainage and right hemicolectomy were also performed in a few cases. The clinical outcomes were promising with uneventful recovery achieved in all patients.

The worldwide incidence of esophageal cancer has markedly increased. Therefore, covered or partially-covered esophageal stents are being increasingly used for luminal patency in patients with the metastatic or inoperable disease, ultimately leading to an increased risk of stent migration. Physicians should maintain a high index of clinical suspicion for serious adverse events like bowel perforation. A close follow-up in patients with esophageal stent placement should be recommended for early recognition of life-threatening complications.

## Conclusions

Small bowel perforation secondary to esophageal stent migration is a rare but potentially life-threatening complication. The diagnosis requires initial clinical assessment in correlation with radiologic findings. Although these patients are usually managed by a case-by-case approach, surgical removal of the migrated stent along with the affected bowel segment followed by end-to-end anastomosis is frequently performed. Physicians should remain vigilant for this unusual sequel to esophageal stent migration as an early detection is vital for appropriate management.
